# UMATBrush: A dataset of inertial signals of toothbrushing activities

**DOI:** 10.1016/j.dib.2025.111980

**Published:** 2025-08-09

**Authors:** F.J. González-Cañete, E. Casilari

**Affiliations:** Departamento de Tecnología Electrónica, Telecommunication Research Institute (TELMA), Universidad de Málaga, 29071 Málaga, Spain

**Keywords:** Inertial sensors, Wearables, Smartwatches, Human activity recognition, Accelerometer

## Abstract

Smartwatches and other commercially available wrist-worn devices have become a low-cost tool which, in recent years, has gained enormous popularity for monitoring habits associated with a healthy lifestyle. In this regard, the increasing computational power of smartwatches is facilitating the integration of complex machine learning and deep learning algorithms, which implement manual activity recognizers based on the inertial sensor signals that these wearables natively include. One specific application of such human activity recognition (HAR) systems is the monitoring of toothbrushing, aimed at fostering oral health habits among the population. For the evaluation and testing of these types of detectors, having access to databases of inertial signals captured by smartwatches is of paramount importance. This work describes the UMATBrush repository, which results from monitoring four experimental subjects during a large number of toothbrushing sessions using three commercial smartwatches. In contrast to other similar repositories, which are focused on the generic development of detectors for a limited set of manual activities, this repository also includes long periods of monitoring of the subjects during their daily lives. In the dataset, each acceleration sample captured by the watches is binary labelled as either corresponding or not to a toothbrushing session. In this way, potential classifiers using these traces could be trained and validated under realistic conditions, by learning to distinguish the toothbrushing operation from other real-life activities.

Specifications Table

**Table:** 

Subject	Computer Sciences
Specific subject area	Human Activity Recognition based on inertial accelerometer signals, identification of toothbrushing sessions using wearable devices.
Type of data	Table, raw text files in CSV format.
Data collection	Four experimental users were monitored with a smartwatch located in the dominant hand during the execution of a series of toothbrushing sessions. In addition, participants were also monitored ‘in the wild’, under real-world conditions, during their daily life routines.
Data source location	The samples were captured in the domestic environments of the four experimental users located in Málaga (Spain) or Rincón de la Victoria (Spain).
Data accessibility	Raw data are available in a public repository hosted by Figshare: Repository name: UMATBrush Data identification number: https://doi.org/10.6084/m9.figshare.28955756 Direct URL to data: https://figshare.com/articles/dataset/UMATBrush_Traces/28955756 Data files can be downloaded from the previous link directly or as a single zip file. A directory named TRACES, with one subfolder for each monitored participant, includes the acceleration data in the form of a set of CSV files. The code of two programs (written in Python and Matlab) are provided in another directory (SCRIPTS) to ease and automate the downloading of the dataset in case it is needed
Related research article	None

## 1 Value of the Data

• The UMATBrush dataset provides accelerometer measurements captured by three different commercial smartwatches and from four participants during the execution of toothbrushing activities and other daily life activities in real-life conditions.
• The dataset can be used to characterize the dynamics of the toothbrushing movements as well as a dataset to train, validate and test AI-based HAR (Human Activity Recognition) systems.
• The dataset offers more acceleration samples of hand movements during toothbrushing than most existing available datasets.
• The number of different sensors (three different smartwatches) also exceeds those considered by other related datasets.
• The dataset may be a helpful tool for the research on broader public health monitoring strategies that leverage wearable inertial data to track daily hygiene routines and to improve patient adherence to preventive care guidelines.
• Potential applications include the development of mobile dental care apps that provide personalized feedback and reminders, as well as remote health-monitoring platforms that use these signals to identify patterns in self-care behaviors relevant to chronic disease management.

## 2 Background

Over the past decade, smartwatches have evolved from fashion accessories to advanced health-monitoring tools with medical-grade capabilities, supporting the detection of sleep quality issues, cardiac arrhythmias, falls, and desaturation events. Their constant, minimally invasive presence can also promote health-driven behavioral changes [1]. In this context, several studies have suggested using smartwatches and smartbands to monitor oral hygiene behaviors (OHB), specifically measuring the duration of toothbrushing sessions via inertial sensors. This is relevant given recommendations to brush for at least two minutes twice daily with fluoride toothpaste, while surveys reveal that nearly 50 % of adults—and over 64 % of those aged 55+—do not brush daily [2]. Developing machine-learning classifiers to detect toothbrushing requires realistic datasets of wrist-based inertial signals. Table 1 summarizes existing related repositories, many of which focus on general human activity recognition (HAR) with limited toothbrushing sessions and other ‘laboratory-generated’ predefined activities. These repositories often lack long-term, real-life traces and use uniform sensor models, limiting generalizability. To address these shortcomings, this work introduces a new dataset of accelerometer signals collected with several commercial smartwatches, capturing both toothbrushing sessions and extended periods of participants’ daily activities to better support OHB detection and HAR system development.

**Table 1:** Existing available datasets with sensor data collected with wrist-worn sensors during toothbrushing activities.

Dataset & reference	No. of subjects	No. of activities	Duration of the samples (s)	Collected signals	Type of device	Sampling rate (Hz)	Model of the sensing node.
Genova [3]	8	17	[3-463]	1 (A)	Sensing mote	252	Actigraph GT9X Link
PAAL [4]	52	24	[0.18-254.59]	1 (A)	Wristband	32	Empatica 4 (E4)
UMAHand [5]	25	29	[1.98-119.98]	4 (A, B, G, M)	Sensing mote	100	SHIMMER 3 mote
Macquaire [6]	22	2	[58.48-364.12]	3(A, G, M)	Sensing mote	200 (A, G), 20 (M)	Mbientlab MetaSensor Mote
HIFD [7]	21	13	[15.72-51.91]	3 (A, G, Q)	Sensing mote	50	E2BOX EBIMU24GV,
Multisensory [8]	10	9	[0.54-9.54]	3 (A, G, Q,)	Sensing mote	33	TSND151
HMP [8]	18	14	[3.87-291.16]	1 (A)	Sensing mote	32	Customized design
WISDM [9]	51	18	[179.68-181.25]	2 (A, G)	Smartwatch	20	, LG G Watch
EJUST-ADL-1 [10]	6	14	[4.26-40.52]	3 (A, G, O)	Smartwatch	50	Apple Watch Series II
VISTA [11]	20	10	[0.9-69.26]	3 (A, G, M)	Sensing mote	100	SensHand Glove
SAMoSA [12]	20	26	[0.4-56.9] s	4 (A, G, M, Mic.)	Smartwatch	50	Fossil Q Explorist Gen 4
University of Texas [13]	15	23	[25.58-55.7]	2 (A, G)	Smartwatch	50	Fossil Q Explorist Gen 4
CogAge [14]	8	7	[2.1-839.26]	2 (A, G),	Smartwatch	100	Huawei Watch

^1^Acronyms for the sensors and magnitudes: A: Accelerometer, B: Barometer, G: Gyroscope, M: Magnetometer, Mic: Microphone, HR: Heart rate, O: Orientation, Q: Quaternion

## 3 Data Description

The database (UMATBrush Dataset [15]) has a main folder and two subfolders named: *Traces* (with the sensed data traces measured) and *Scripts*. The first one contains the collected accelerometer data while the second one includes two scripts that automatically download, unzip and process the dataset.

The *Traces* subfolder in turn consists of four subfolders, each corresponding to one of the experimental subjects. These are named with the word *Subject*, followed by an underscore (_) and the subject’s identifier (a number from 1 to 4).

The names of the monitored data files with the acceleration measurements are presented according to the layout *Subject_X_Watch_WWW_Date_YYYYMMDD_Time_HHMMSS.csv,* where:

• *X* is the subject identification number (1 to 4).
• *WWW* is the smartwatch model name used to capture the acceleration signals. It can take one of the following values: LEO-DLXX, TicWatch-Pro or TicWatch-Pro-3-GPS, as indicated in the next section.
• *YYYYMMDD* is the year (*YYYY*), month (*MM*) and day (*DD*) when the trace was collected.
• *HHMMSS* is the hour (*HH*), minute (*MM*) and second (SS) of the instant when the gathering of the samples was initiated.

Data files are plain text files in CSV (Comma Separated Values) format without headers. Every line in each data file contains a single measurement of the triaxial accelerometer of the IMU embedded in the smartwatch. The values of each line are arranged as it follows:

• ***Timestamp, Ax, Ay, Az, Class Label***

where:

***Timestamp*** is the time (in μs) elapsed from the starting time of the recording to the instant in which the sample was captured. Accordingly, the first sample has a zero value and the rest of samples have a timestamp relative to this first sample.

***Ax, Ay, Az*** are the measurements of the three axes of the triaxial accelerometer (expressed in g units).

***Class Label*** is a binary value that indicates whether the sample corresponds to a toothbrush activity (1) or not (0).

Finally, the *Scripts* subfolder includes two programs respectively developed in Python and Matlab. Both scripts, which are named *Load_traces*, have the same purpose: to automate the process of downloading and handling the dataset. Specifically, they perform the following operations:

1. Retrieve the dataset from the public repository as a single compressed ZIP file.
2. Extract the contents of this file and set up the dataset’s subfolder structure within a designated main directory called *UMATBrush_Dataset*.
3. Read all the CSV files and store their contents in a data structure: a list of dictionaries in Python or a matrix of structures in Matlab, referred to as *datasetTraces*. Each element in this list or matrix includes two fields: the filename (which identifies the user, smartwatch and date and time of the experiment) and a numerical array with five columns containing: the timestamp, the three measurements captured by the triaxial sensor and, finally, the label for the measurement (binary formatted as previously described).

## 4 Experimental Design, Materials and Methods

Although the repositories mentioned in the Background section are undoubtedly of great interest, none of them was specifically designed for the development of OHB tracking systems, but rather for the design of general (multi-activity) HAR systems focused on hand movements. As a result, the number of toothbrushing sessions included is quite limited. Additionally, these datasets were created based on a structured, predefined and limited set of (manual or non-manual) activities that participants were required to perform repeatedly and under controlled laboratory conditions. Therefore, they do not include long-term traces captured in real-life scenarios, with unexpected or spontaneous movements that could pose a challenge for the training and testing of classifiers as they were not contemplated in the restricted list of activities executed by the participants. Besides, the sensors used in a dataset’s testbed may introduce biases or specific characteristics that affect the measurements. In this sense, another limitation of the existing repositories is that they use the same sensor (or the same sensor model) for all wrist-based measurements. In contrast, it would be valuable to have traces collected using a variety of devices, which would allow, for instance, the evaluation of a model’s ability to generalize when trained and tested on data obtained from different sensor models or vendors. Consequently, the main goal of the data collection in our testbed was to support practical applications in oral health monitoring by capturing realistic, real-world traces with several commercial smartwatches acting as sensing tools.

### 4.1 Sensing devices

Three different Android Wear OS based smartwatches were employed to create the dataset. Table 2 lists their main characteristics.

**Table 2:** Specifications of the smartwatch models used for data collection.

	Huawei Watch 2 [16]	Mobvoi Ticwatch Pro 2020 [17]	Mobvoi Ticwatch Pro 3 [18]
Android Wear OS version	2.44 based on Android 8.0	2.44 based on Android 9.0	2.44 based on Android 9.0
Microprocessor	Qualcomm Snapdragon 2100	Qualcomm Snapdragon 2100	Qualcomm Snapdragon 4100
RAM memory	768 MB	1 GB	1 GB
Storage capacity	4 GB	4 GB	8 GB
Battery	420 mAh	415 mAh	577 mAh
Accelerometer sensor	STMicroelectronics LSM6DS3	STMicroelectronics LSM6DSM	STMicroelectronics LSM6DSO
Max sensor range	±4 g	±8 g	±8 g
Watch identifier in the traces	LEO-DLXX	TicWatch-Pro	TicWatch-Pro-3-GPS

A Wear OS application was developed and installed in the smartwatches in order to capture the subject’s movements. The application incorporates a button that triggers the measurement of the movements by capturing the values returned by the built-in accelerometer and storing them in text files in the smartwatch internal memory. In order to label the traces accordingly as toothbrush or non-toothbrush, once the capturing process has been initiated, a second on/off button enables the user to specify the initial and final moments of each toothbrush session.

After the monitoring was complete, the raw files stored in the smartwatches were transferred to a personal computer and subsequently post-processed using the MATLAB programming environment [19] in order to produce the final data format employed in the dataset and described in the preceding section.

### 4.2 Protocol to collect the measurements

With the aim to construct the dataset, three volunteers were recruited: two female and one male, with an average age of 49 years. All participants reported not suffering from any specific dental pathologies and were not undergoing any form of orthodontic treatment at the time of the monitoring campaign. Each of the three volunteers (all of whom were right-handed) was assigned a different smartwatch model.

During the initial phase of data collection, volunteers were instructed to wear the smartwatch immediately upon waking up and to initiate the measurement process without delay. Hence, the smartwatches recorded extended acceleration traces under real-world conditions, capturing data throughout the participants' daily routines. The experimental subjects were also asked to manually indicate the start and end of each toothbrushing session by pressing the designated on/off button on the smartwatch just before (and after) the beginning (and end) of the toothbrushing activity. Depending on the state of that button, the app was responsible for adding the corresponding binary label to each recorded sample gathered from the triaxial accelerometer in the flash (non-volatile) memory of the watch. In all cases, data collection continued uninterrupted until the smartwatch battery was fully depleted.

In a subsequent phase, to increase the number of toothbrushing-specific traces, participants were requested to wear the smartwatch and initiate the data recording exclusively during additional toothbrushing sessions.

Finally, a fourth participant (male, aged 49) was also recruited. This volunteer was asked to perform the same two monitoring phases of data collection as the previous volunteers but now alternatively using the three smartwatches.

Throughout the entire monitoring campaign, no user reported experiencing any discomfort or indicated that they had altered their hygiene habits as a result of wearing the smartwatch.

During the execution of each activity, each experimental subject wore the corresponding smartwatch adjusted to the wrist of the dominant hand. For all the experiments, the sampling frequency configured in the watches was set to an approximate value of 100 Hz. For that purpose, the so-called SENSOR_DELAY_FASTEST option offered by the Wear OS API was set in the app. This constant obliges the wearables to capture the samples of their embedded sensors at the highest update frequency.

Power spectrum of hand movements during toothbrushing concentrates around 4-5 Hz, with just some particular actions that can involve components higher than 10 Hz [20]. Thus, a sampling frequency of 100 Hz is more than adequate to capture and characterize the dynamics of hand motion with high fidelity.

All experiments and data acquisition were conducted in the private home environments of the participants without any kind of direct supervision of the researchers.

To conclude this subsection, Table 3, Table 4 respectively describe the duration of the samples recorded with each smartwatch and the number of complete toothbrushing sessions monitored for each experimental subject.

**Table 3:** Duration of the traces collected with each smartwatch model used in the testbed.

	Huawei Watch 2	Mobvoi Ticwatch Pro 2020	Mobvoi Ticwatch Pro 3
**Toothbrushing traces**	1h 9’ 43’’ (4,183 s)	1h 55’ 17’’ (6,917 s)	2h 8’ 5’’ (7683 s)
**Traces of other routines**	23h 49’ 25’’ (85,765 s)	101h 56’ 09’’ (366,969 s)	116h 11’ 26’’ (418,286 s)

**Table 4:** Number of toothbrushing sessions during which each experimental subject was monitored.

	Subject #1	Subject #2	Subject #3	Subject #4
Toothbrushing sessions	29	31	45	39

## Limitations

The hand used by conventional users to handle the toothbrush is almost always the dominant hand; therefore, this location—unusual or unnatural for wearing a watch—was chosen to place the smartwatch worn by the participants during data collection. Besides, the study was limited to manual toothbrushing sessions, which remains the most common method for this oral hygiene routine. Future versions of the repository should consider capturing samples while employing electric toothbrushes, a tool recommended by many dentists and which is gradually attracting more attention among users.

Another limitation of this dataset is its small sample size, restricted to four participants. This reduced number may affect the generalizability of the recorded traces, suggesting caution when applying trained models to broader or more diverse populations. In this respect, expanding the participant pool to cover different age groups and dental conditions would help improve the representativeness and applicability of the dataset.

## Ethics Statement

The experimental procedure conformed to the ethical guidelines of the authors’ affiliated institutions and received formal approval from the Ethics Experimentation Committee of the University of Málaga (CEUMA) during its session held on October 31, 2024, in response to the submitted project proposal registered under reference number 158-2024-H. Prior to participation, all volunteers provided written informed consent in compliance with European Regulation 2016/679, commonly known as the General Data Protection Regulation (GDPR).

Participants were also provided with a detailed information sheet outlining the objectives and methodology of the study, as well as the nature and types of data to be collected. They were explicitly informed of their right to withdraw from the study at any point during the data acquisition process, without the need to provide justifiction and without any repercussions.

## CRediT authorship contribution statement

**F.J. González-Cañete:** Conceptualization, Methodology, Software, Investigation, Data curation, Writing – original draft, Writing – review & editing. **E. Casilari:** Conceptualization, Methodology, Supervision, Writing – original draft, Writing – review & editing, Project administration, Funding acquisition.

## Data Availability

FigshareUMATBrush (Original data)
